# Autophagic flux is highly active in early mitosis and differentially regulated throughout the cell cycle

**DOI:** 10.18632/oncotarget.9451

**Published:** 2016-05-18

**Authors:** Zhiyuan Li, Xinmiao Ji, Dongmei Wang, Juanjuan Liu, Xin Zhang

**Affiliations:** ^1^ High Magnetic Field Laboratory, Chinese Academy of Sciences, Hefei, Anhui, 230031, P. R. China; ^2^ University of Science and Technology of China, Hefei, Anhui, 230036, P. R. China

**Keywords:** mitosis, autophagy, autophagic flux, LC3, cell synchronization

## Abstract

Mitosis is a fast process that involves dramatic cellular remodeling and has a high energy demand. Whether autophagy is active or inactive during the early stages of mitosis in a naturally dividing cell is still debated. Here we aimed to use multiple assays to resolve this apparent discrepancy. Although the LC3 puncta number was reduced in mitosis, the four different cell lines we tested all have active autophagic flux in both interphase and mitosis. In addition, the autophagic flux was highly active in nocodazole-induced, double-thymidine synchronization released as well as naturally occurring mitosis in HeLa cells. Multiple autophagy proteins are upregulated in mitosis and the increased Beclin-1 level likely contributes to the active autophagic flux in early mitosis. It is interesting that although the autophagic flux is active throughout the cell cycle, early mitosis and S phase have relatively higher autophagic flux than G1 and late G2 phases, which might be helpful to degrade the damaged organelles and provide energy during S phase and mitosis.

## INTRODUCTION

Mitosis is an important cellular event where a cell segregates its chromosomes and cellular contents into two daughter cells. The mitotic phase is much shorter compared to other phases in the whole cell cycle. However, it is highly dynamic that includes prophase, prometaphase, metaphase, anaphase, telophase and cytokinesis. All these dynamic events need to be finished in 1–2 hours, which is a high energy demanding process and involves lots of cellular remodeling [1–4].

Although it was demonstrated by multiple studies that autophagy plays an important role in cytokinesis [5, 6], the last step of mitosis, whether autophagy persists during the early phases of mitosis is still a debate [7]. A few studies indicated that autophagy was inhibited during mitosis [8, 9]. In 2002, Eskelinen et al. used morphological studies to show that autophagic vacuole number was decreased in nocodazole-arrested cells as well as in cells just released from nocodazole [8]. In 2010, Furuya et al. found that key mitotic kinases Cdk1 and Cdk5 phosphorylated VPS34 during mitosis, which disassociated VPS34 from its partner Beclin-1. Therefore they proposed that the inhibition of VPS34 during mitosis might suppress autophagy to protect the fragmented Golgi during mitosis [9, 10].

At the same time, there were also some evidences indicating that autophagy may persist during mitosis [11, 12]. In 2009, Liu et al. tried to address this question directly by using autophagic flux inhibitors to block autophagic flux and they found that the autophagosomes can be accumulated in mitotic cells [11]. This indicated that autophagy is active in mitosis. However, several questions arise from their study. First of all, they treated cells with inhibitors overnight, by which the accumulated autophagosomes in the harvested mitotic cells could be inherited from the interphase cells. They also used ammonia as an autophagic flux inhibitor, which was found to function as an autophagy inducer in addition to its role as an inhibitor [13–16]. Therefore the observed LC3BII increase can potentially be a result of NH_4_Cl-induced autophagy. In addition, nocodazole depolymerizes microtubules, which could potentially affect autophagy as well. In a recent study, Domenech et al. found that prolonged mitotic arrest induces an early autophagic flux response that results in mitochondrial degradation by autophagy [12]. This is a very important finding, which demonstrates that the mitotic arrested cells have an active autophagic flux. However, whether the naturally occurring mitotic cells have active autophagic flux was still unclear.

It has been reported by Tasdemir et al. that different autophagy inhibitors/inducers can cause differential effects in a cell cycle-dependent way [17]. However, Tasdemir et al. used GFP-LC3 puncta to indicate autophagic structures, which is not always reliable because the GFP fluorescence can be quenched in acidic vesicles. A recent study used anti-LC3 antibody staining in flow cytometry to monitor the autophagy level in different stages of cell cycle by measuring LC3II level and found that autophagy activity was at the same level in G2/M, S or G0/G1 phases [18]. Although this method ruled out the possibility of GFP fluorescence quenching, this flow cytometry method could not separate mitosis from G2 phase.

Whether autophagy shuts down or persists during naturally occurring mitosis, especially during the earlier stages of mitosis, is still an open question. Here in this study, we examined autophagic flux in four different cell types. In addition, we tested HeLa (human cervical cancer) cells in nocodazole-induced, double-thymidine synchronization released, and naturally occurring mitosis. More importantly, we also compared the autophagic flux in different stages during mitosis and the whole cell cycle. Our results show that autophagic flux is active throughout the cell cycle, but is more active in S phase and early mitosis compared to G1, G2 and late mitosis.

## RESULTS

### Autophagy is active in naturally occurring mitotic cells in four different cell lines

**Figure 1:**
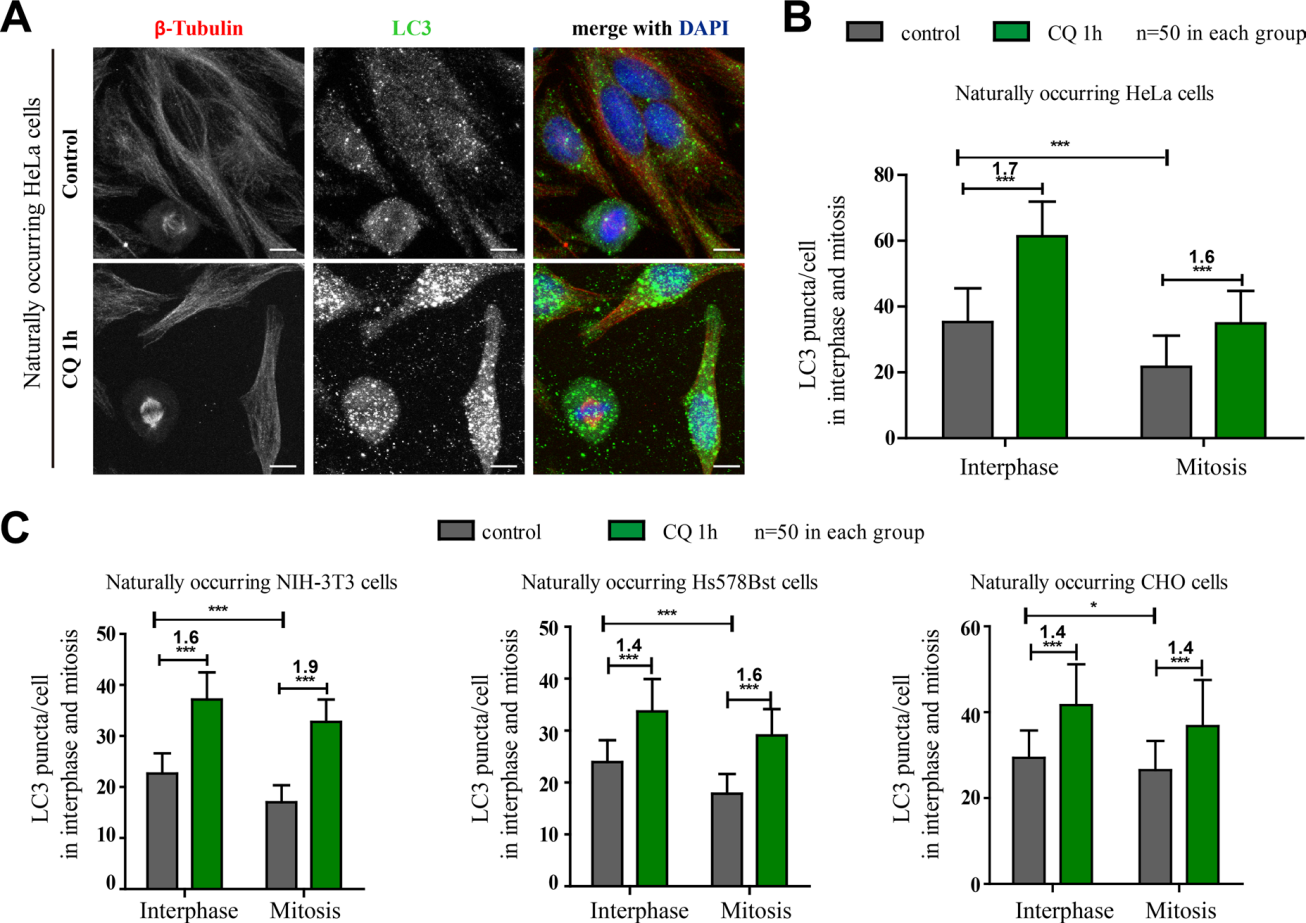
Autophagy is active in naturally occurring mitotic cells in four different mammalian cell lines (**A**) Immunofluorescence results showed that autophagy was active in unperturbed mitotic HeLa cells. HeLa cells were treated with control or 25 μM chloroquine (CQ) for 1 hour and autophagy levels were determined by co-stained with anti-β-Tubulin(red) and anti-LC3 (green) antibodies. Cells were fixed with −20°C methanol for 5 minutes and then subjected to immunostaining assay. Scale bar, 10 μm. (**B**) Quantification of LC3 puncta per HeLa cell showed the fold change of LC3 puncta/cell after chloroquine treatment in interphase or mitosis. (**C**) Quantification of LC3 puncta per cell in three different non-cancer cell lines with or without chloroquine treatment. 3T3, Hs578Bst and CHO cells were treated with control or 25 μM chloroquine (CQ) for 1 hour and autophagy levels were determined by co-stained with anti-β-Tubulin(red) and anti-LC3 (green) antibodies. Cells were fixed with −20°C methanol for 5 minutes and then subjected to immunostaining assay. 50 cells were counted for each condition. *p < 0.05; ***p < 0.001.

The previously reported studies related to autophagy and mitosis mostly used cancer cell lines, and multiple of them used HeLa cells, a human cervical cancer cell line. Therefore we also tested HeLa cells first in our study. Multiple studies concluded that autophagy was inactive in mitosis because mitotic cells had less autophagic structures, which we also observed (Figure 1A). However, decreased autophagic structure number does not necessarily mean that the autophagy is inhibited. It could also be a result of rapid clearance of autophagosomes due to an elevated autophagic flux. To evaluate the autophagic flux status, comparing the autophagosomes change by adding an autophagic flux inhibitor such as chloroquine, a small molecule that prevents intravesicular acidification, is a frequently used method [19]. At the same time, since we are investigating mitosis, a highly active process, we tried to avoid long incubation time of autophagic flux inhibitor to minimize the possibility of inherited autophagosomes from earlier cell cycle stages. Here we used chloroquine instead of NH_4_Cl to avoid any autophagy inducing effects. Our results showed that the autophagosomes number in both interphase and mitotic cells was obviously increased after chloroquine treatment in HeLa cells (1.7-fold increase in interphase cells and 1.6-fold increase in mitotic cells, *p* < 0.001). The ATG12 staining in mitotic HeLa cells was also increased after chloroquine treatment (Figure S1). In addition, we also imaged the live mCherry-EGFP-LC3-HeLa cells and found that although the green LC3 puncta were greatly reduced because of the acidic quenching of GFP, the red LC3 puncta (mCherry is not pH sensitive) were robustly present in mitotic cells (Figure S2). These results indicate that the autophagic flux is active in both interphase and mitosis (Figure 1A–1C).

To exclude the possibility that the phenotype we observed was HeLa-specific, we also tested three non-cancer cell lines, 3T3 (a mouse embryo cell line), Hs578Bst (a human breast cell line) and CHO (a Chinese Hamster Ovary cell line). Immunofluorescence experiments show that they all have reduced LC3 puncta in mitosis (Figures 1C, S3). Consistent with the results of HeLa cells, the autophagosomes numbers in both interphase and mitotic cells were also obviously increased after chloroquine treatment in these three cell lines (Figure 1C). All these evidences above suggest that autophagy is active in mitotic cells.

### Autophagy is active in nocodazole-induced mitosis

**Figure 2:**
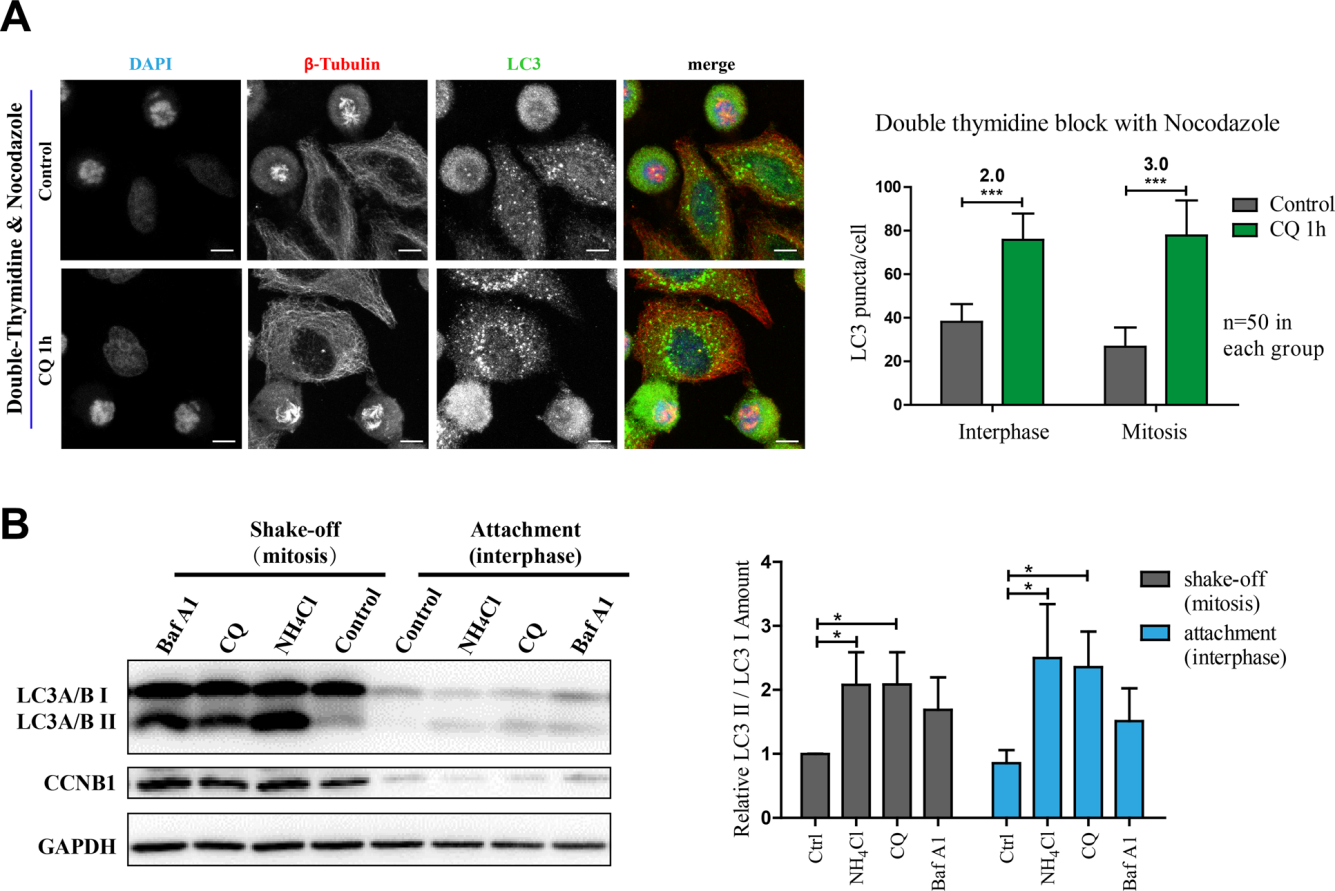
Autophagy is active in nocodazole-induced mitosis (**A**) Immunofluorescence results show that autophagy is active in nocodazole-induced mitotic cells. Autophagy levels were determined by co-stained with anti-β-Tubulin (red) and anti-LC3 (green) antibodies in nocodazole-induced mitotic cells. Nocodazole-induced mitotic cells were acquired with double-thymidine block combined with 4 hours release and 7 hours nocodazole treatment and treated with control or 25 μM chloroquine (CQ) for 1 hour. Cells were fixed with −20°C methanol for 5 minutes and then subjected to the following immunostaining assay. Scale bar, 10 μm. Left panel shows the immunofluorescence results and right panel shows the quantification of LC3 puncta fold change per interphase or mitotic cell after chloroquine treatment. (**B**) Western Blot results show that autophagy in mitotic cells is active. Nocodazole-induced mitotic cells were treated with indicated concentrations of autophagy disrupters (10 mM NH_4_Cl, 25 μM chloroquine and 20 nM bafilomycin A1) for 1 hour. Mitotic and interphase cells were separated with shake-off. Cells were lysed to perform Western Blot analysis. Representative results for anti-LC3 A/B, anti-Cyclin B1 (CCNB1) and anti-GAPDH were shown in left panel. Quantification of relative LC3 II/LC3I amount is shown in right. Data show mean ± SD. for three independent experiments. *p < 0.05; ***p < 0.001. Representative results were shown in the figure.

We next determined that if the autophagic flux is active in nocodazole-induced pseudo-mitosis (prometaphase-like), a commonly used method to investigate mitosis. We treated cells acquired from double-thymidine and nocodazole synchronization with or without chloroquine before they were harvested for immunofluorescence experiments. Our results showed that the LC3 puncta number in both interphase and mitosis was increased to the similar extent after 1 hour chloroquine treatment, indicating that the autophagic flux was active in all phases of cells after nocodazole treatment (Figure 2A). We also used Western Blot analysis in a shake-off experiment to compare the autophagic flux in mitotic (shake-off) and interphase (attachment) cells. The high expression of Cyclin B1 in the shake-off cells verified their early mitotic stage (Figure 2B). It was evident that the overall LC3 as well as LC3II/I were elevated in mitosis. In addition, we used three different chemicals, NH_4_Cl, chloroquine and bafilomycin A1 (an inhibitor of vacuolar H (+)-*ATPases*), to block autophagic flux and compared the fold change. It is obvious that the LC3II/I ratio was effectively increased by all these autophagic flux inhibitors, in both mitotic and interphase cells (Figure 2B). These results showed that autophagic flux was active in nocodazole-induced pseudo-mitosis.

### Autophagic flux is active in mitotic cells released from double thymidine block

**Figure 3:**
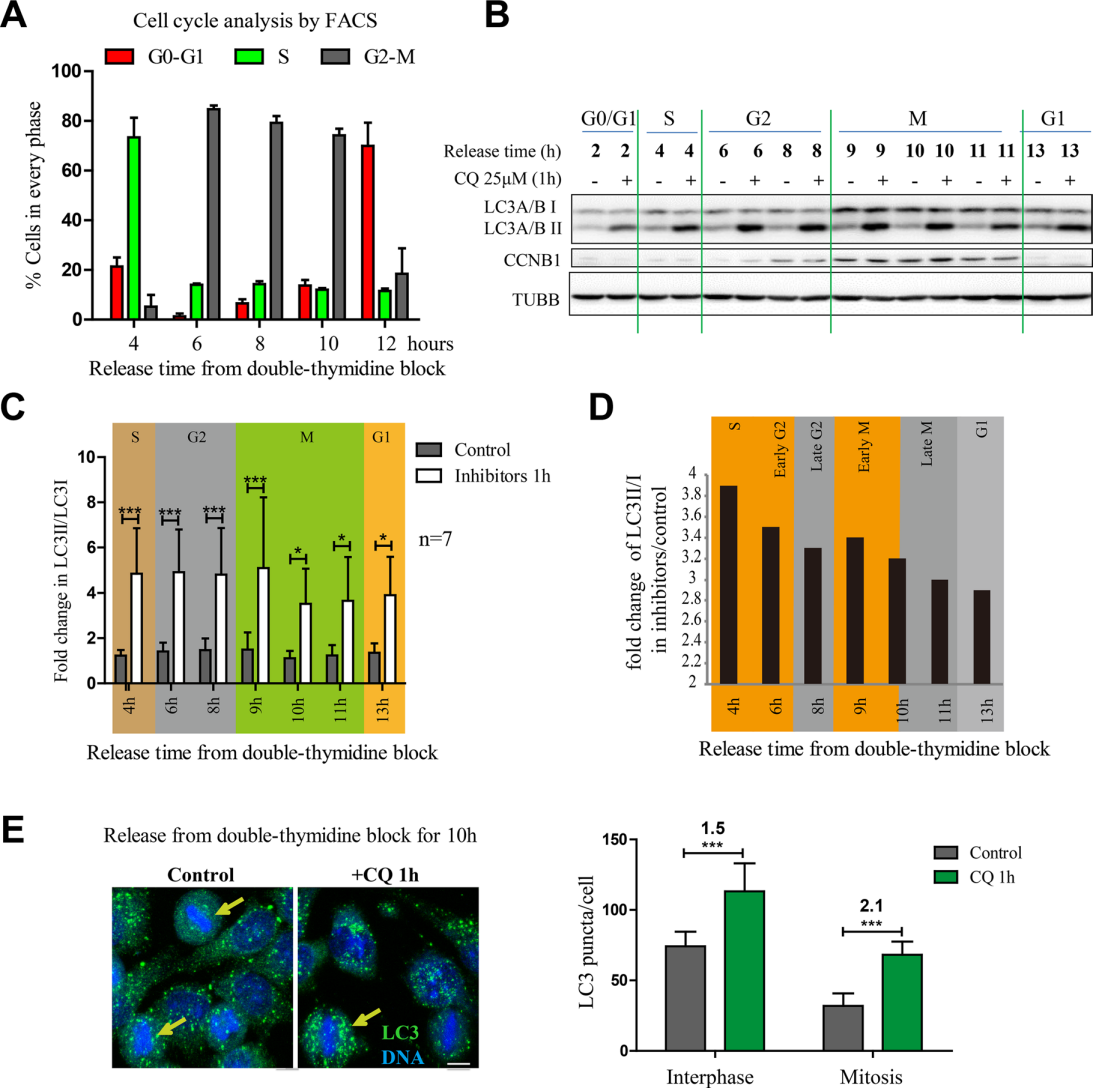
Autophagic flux is active in mitotic cells released from double thymidine block (**A**) Quantification of FACS results that determined the HeLa cell cycle distribution of cells released from double-thymidine block at different timepoints. *n* = 3. (**B**) Western Blot analyzed the autophagy level of HeLa cells released from double-thymidine block. HeLa cells were released from double-thymidine at different timepoints and treated with 25 μM chloroquine for 1 hour. Representative results were shown with anti-LC3 A/B, anti-Cyclin B1 (CCNB1) and anti-Tubulin antibodies. (**C**) Quantification of results in (B) shows fold change in LC3 II/LC3I. (**D**) Quantification of results in (B) shows fold change of LC3 II/LC3I in chloroquine/control group. (**E**) Immunofluorescence analyzes the autophagy level of HeLa cells released from double-thymidine block. HeLa cells were released from double-thymidine block for 10 hours, treated with or without 25 μM chloroquine for 1 hour and fixed with −20°C methanol and stained with anti-LC3 A/B antibody (green) and DAPI (blue). The arrow (yellow) indicates the mitotic cell. Scale bar, 10 μm. Bottom shows the quantification of LC3 puncta per interphase or mitotic cell with or without chloroquine treatment. The numbers show the fold change after chloroquine treatment. Representative results were shown in the figure. Data show mean ± SD. for at least three independent experiments. *p < 0.05, **p < 0.01, ***p < 0.001.

To reduce the possibility of nocodazole-induced artifact, we used another commonly used method, double thymidine block, to synchronize cells. Double thymidine block arrests cells in G1/S boundary and release the block will enable cells progress through S phase, G2 phase and mitosis. Although the cells are no longer well synchronized 24 hours after release (Figure S4), the synchronization efficiency is good from 2–12 hours (Figures 3A and S4) and usually cells can enter mitosis at around 8–9 hours after double thymidine release (Figure 3A). To analyze the detailed changes of autophagic flux during the cell cycle, we treated cells with double thymidine, release the synchronization and added chloroquine for 1 hour before we collected cells at different time points. The total LC3 level was increased during mitotic stage (Figure 3B, 3C), which was consistent with the results we got in nocodazole-induced pseudo-mitosis. In addition, chloroquine could effectively increase the LC3II/I throughout the cell cycle (Figure 3D), which indicates that the autophagic flux is active throughout the cell cycle. Immunofluorescence experiment also confirmed that the mitotic cells that we collected after double thymidine synchronization had active autophagic flux because 1 hour of chloroquine treatment effectively increased the LC3 puncta (Figure 3E).

### LC3B and Beclin-1 are upregulated in mitosis

**Figure 4:**
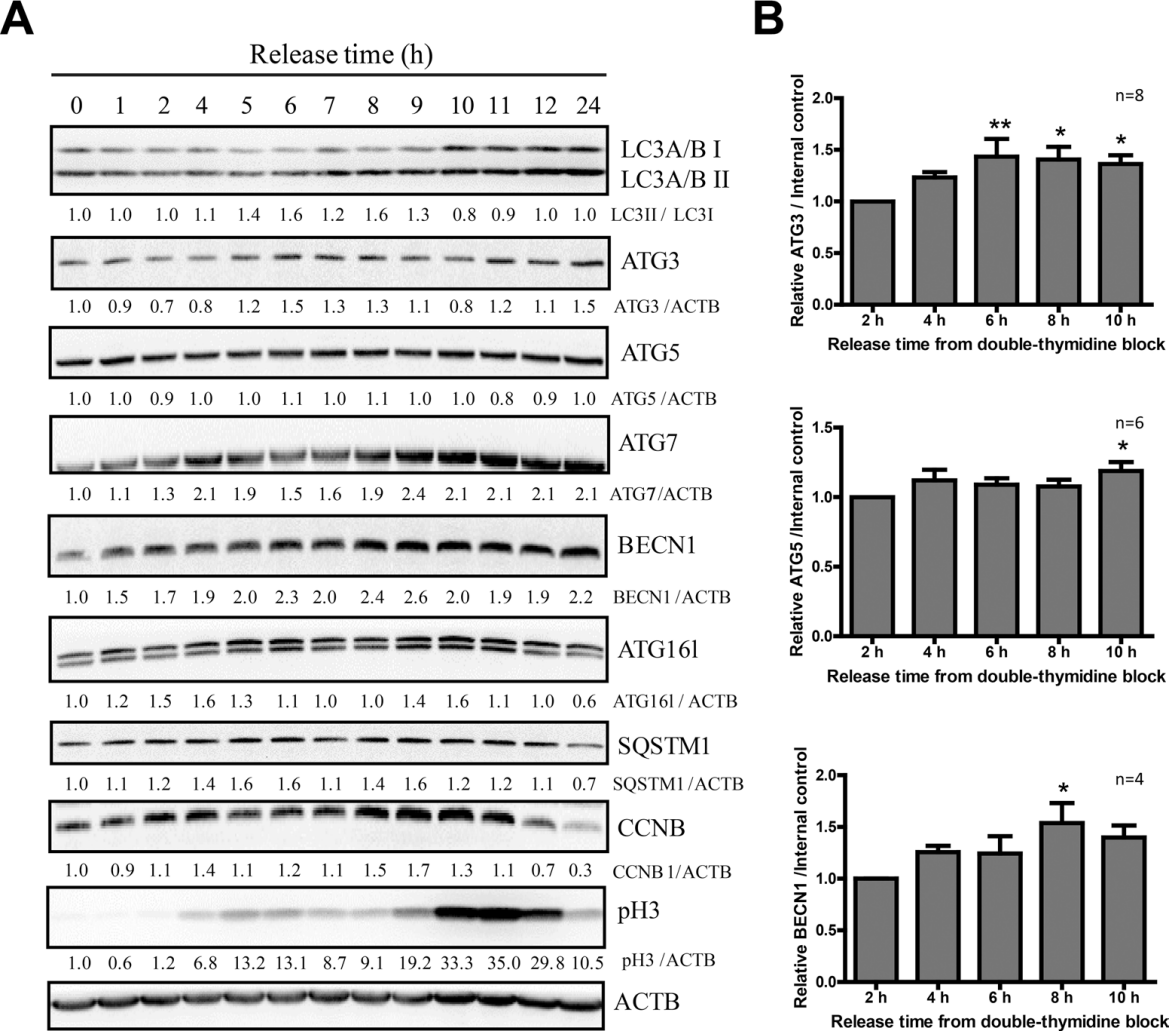
LC3B and Beclin-1 protein levels are upregulated in mitosis (**A**) Western Blot analyzed the autophagy protein levels of HeLa cells released from double-thymidine block. HeLa cells were released from double-thymidine at different timepoints. Densitometric analysis was performed and quantification results were labeled below the corresponding blots. Experiments were repeated for more than 3 times and representative results are shown. (**B**) Quantification of ATG3, ATG5 and Beclin-1 in (A) from 4–8 independent experiments. Comparisons between different timepoints were analyzed by one-way ANOVA followed by Dunnett's Multiple Comparison Test using 2 hours as standard. Data show mean ± SEM. *p < 0.05, **p < 0.01.

The above experiments so far demonstrated that the autophagic flux in mitotic HeLa cells collected through three different methods, including naturally occurring, nocodazole-induced, and double thymine released, was all active. Next we investigated some autophagy protein levels in mitosis since we noticed an increase of the LC3 total protein level in mitotic cells (Figures 2B and 3B). We used double thymidine to synchronize HeLa cells and release them for different time points before they were harvested for western blots to analyze some key autophagy protein levels. We notice that not only LC3 protein, but also a few other proteins are upregulated in mitosis, such as ATG3, ATG5 and Beclin-1 (Figure 4A, 4B).

**Figure 5:**
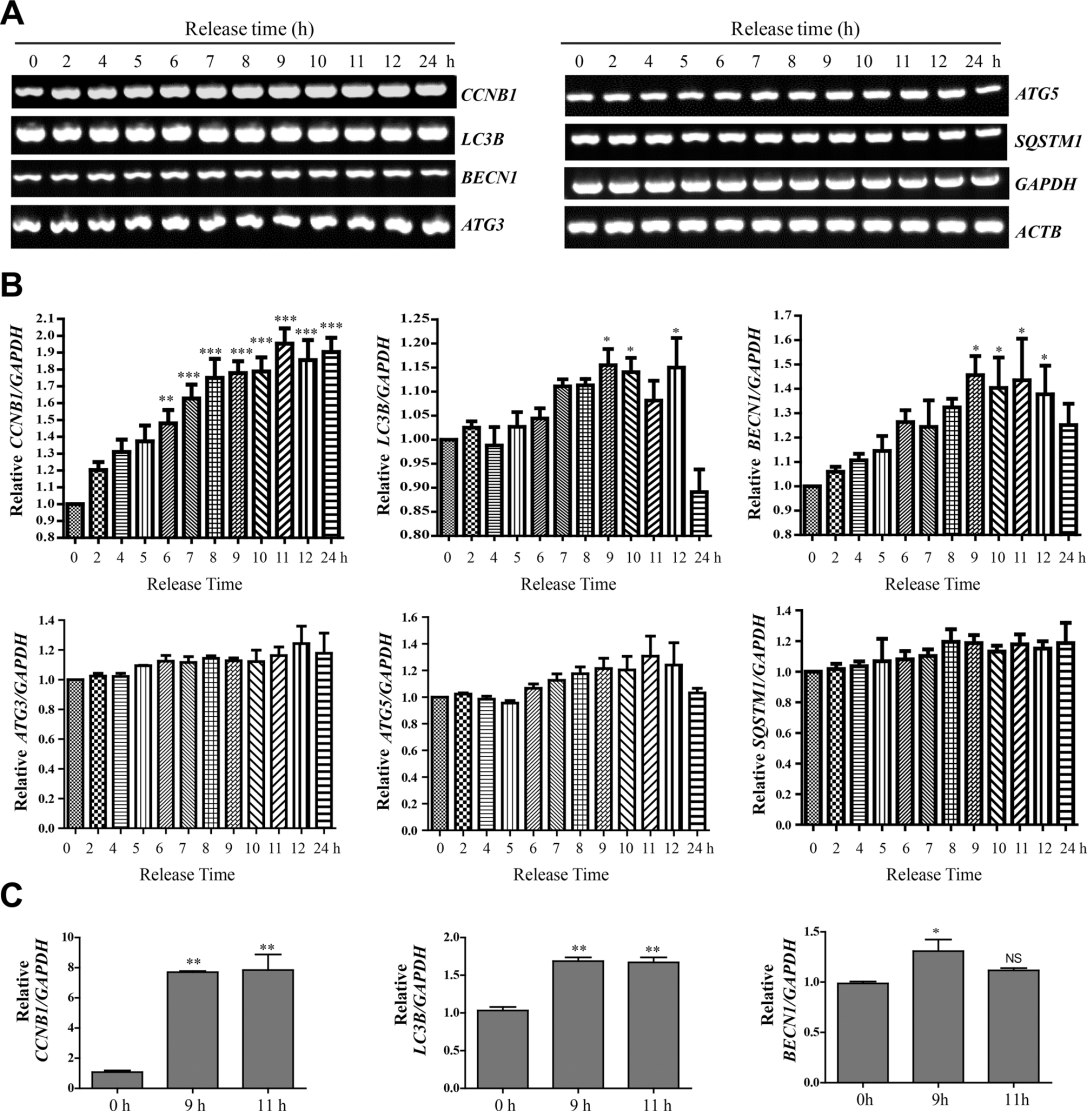
LC3B and Beclin-1 mRNA levels are upregulated in mitosis (**A**) Semi-quantitative RT-PCR of CCNB1 (Cyclin B1), LC3B, BECN1 (Beclin-1), ATG3, ATG5, SQSTM1, GAPDH and ACTB. (**B**) Quantification of (A). Data show mean ± SEM. for three independent experiments. The values were normalized to GAPDH. Comparisons between different timepoints were analyzed by one-way ANOVA followed by Dunnett's Multiple Comparison Test using 0 hour as standard. *P* values are labeled in the figures for where data were compared. *p < 0.05, **p < 0.01, ***p < 0.001. (**C**) Real-time RT-PCR of CCNB1 (Cyclin B1), LC3B, BECN1 (Beclin-1) and GAPDH. Quantification shows the relative amount of CCNB1, BECN1 and LC3B (normalized to GAPDH). Comparisons between different timepoints were analyzed by one-way ANOVA followed by Tukey's Multiple Comparison Test using 0 hour as standard. Data show mean ± SD. *p < 0.05, **p < 0.01.

We then used semi-quantitative RT-PCR to analyze the mRNA of these autophagy-related genes (Figure 5A, 5B) in different cell cycle stages, using Cyclin B1 (CCNB1) as a cell cycle marker. We found that at mRNA level, both Beclin-1 and LC3B were upregulated in mitosis, while the changes in the other autophagy genes were not significant (Figures 5B). The increased ATG3 and ATG5 protein level (Figure 4) may not only due to the mRNA change, but also decreased protein degradation in mitosis. In addition, we further confirmed the LC3B and Beclin 1 mRNA level increase by real-time RT-PCR (Figure 5C). The increased Beclin-1 in mitosis indicates that it may have some mitotic function, which is consistent with a previous report that Beclin-1 knockdown caused mitotic arrests and chromosome misalignment [20].

### S phase and early mitosis have higher autophagic flux than the other cell cycle stages

**Figure 6:**
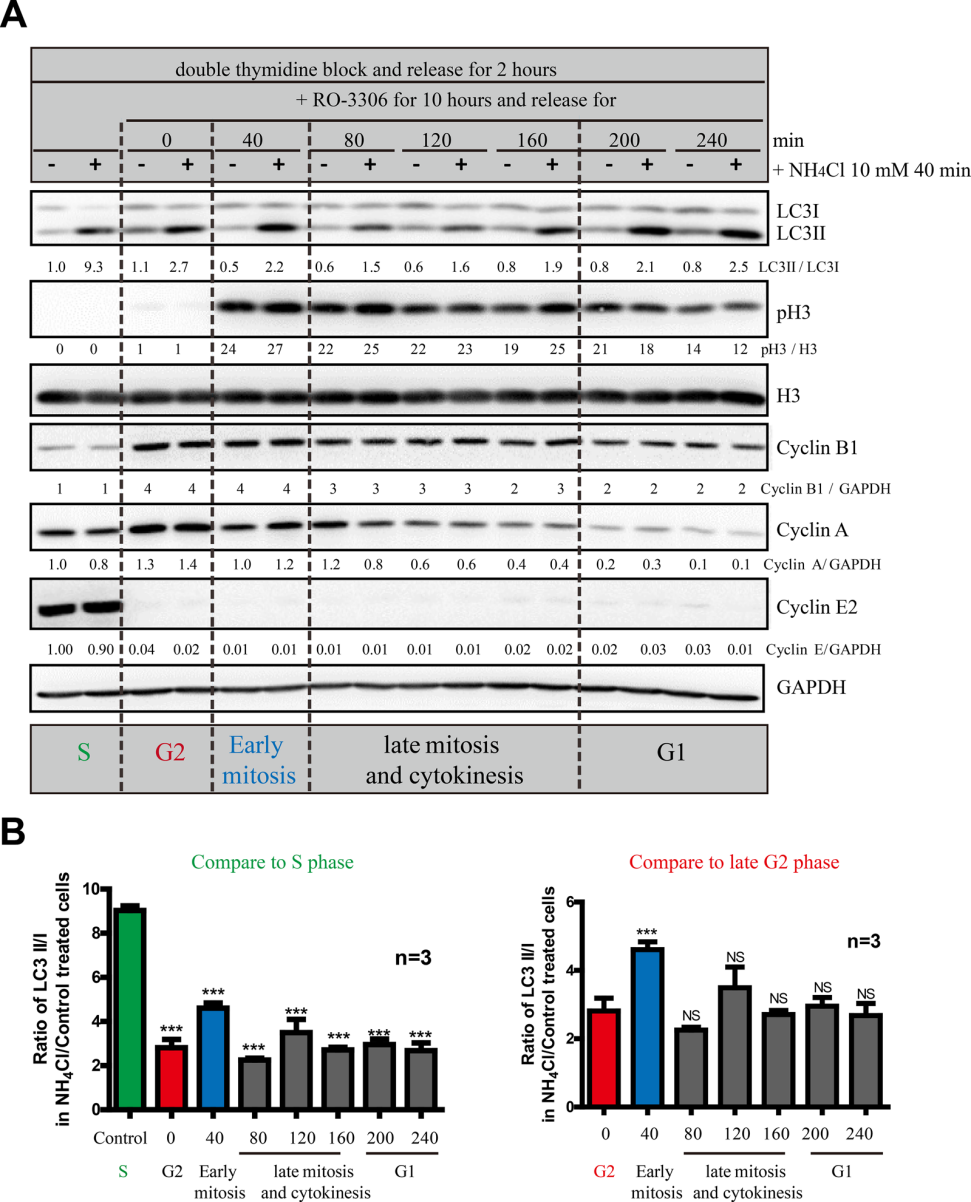
S and early mitosis have higher autophagic flux than the other cell cycle stages (**A**) Western Blot analyzed the autophagic flux of HeLa cells released from double-thymidine with or without RO-3306 synchronization in combination with NH_4_Cl. HeLa cells were released from double-thymidine for 2 hours before they were treated with RO-3306 for 10 hours. Then they were released for different timepoints before they were treated with or without 10 mM NH_4_Cl for 40 minutes. Densitometric analysis was performed and quantification results were labeled below the corresponding blots. Experiments were repeated for three times and representative results are shown. (**B**) Quantification of (A). Left histogram uses S phase as standard in statistic analysis and the right histogram uses G2 phase as standard. Data show mean ± SD. for three independent experiments. Comparisons were analyzed by one-way ANOVA followed Dunnett's Multiple Comparison Test. ***p < 0.001; NS, not significant.

Next we wanted to further dissect the autophagy level at different cell cycle stages, especially mitosis. Although double thymidine block is a commonly used synchronization method, it does not provide accurate temporal dissection for G2 and mitosis. Here we used RO-3306, a CDK1 inhibitor that synchronized cells at late G2 phase [21, 22]. Upon release from RO-3306, cells go through mitosis step by step. Using some cell cycle markers, such as Cyclin A, Cyclin E, Myt1, p21 and phosphor-Histone 3, we show that we can clearly dissect the S phase, G2 phase, early and late mitosis, as well as G1 phase using a combination of double thymidine and RO-3306 (Figure 6A). In addition, to analyze the autophagic flux, we used NH_4_Cl instead of chloroquine, bafilomycin A1 or E64d/Pepstatin (lysosomal proteases inhibitors) as an autophagic flux inhibitor because NH_4_Cl can work faster (40 minutes) than the others (at least one hour), which is crucial for investigating the fast mitosis process. By comparing the LC3II/I level in the presence or absence of NH_4_Cl, we found that although autophagic flux is constantly active throughout the whole cell cycle, S phase has the highest level (Figure 6). Other than S phase, early mitosis has a higher autophagic flux compared to late G2, late mitosis and G1 phase.

### Autophagic flux is active throughout interphase and mitosis and is highly active during early mitosis in unperturbed cells

**Figure 7:**
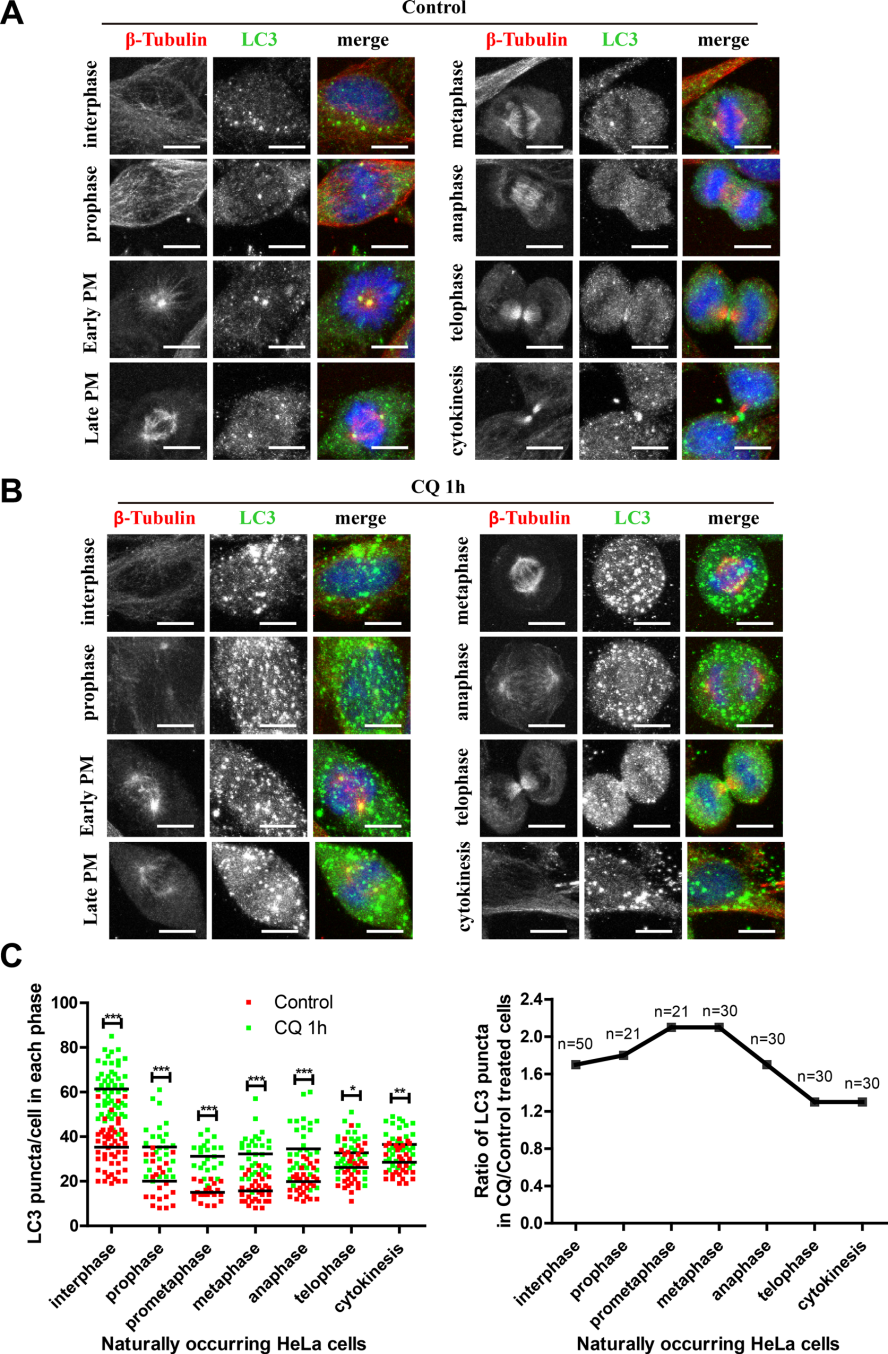
Autophagic flux is active throughout mitosis and is highly active during early mitosis in unperturbed cells (**A**) Autophagy level was equivalent in every phase of naturally occurring cells. (**B**) Autophagy level was equivalent in every phase of naturally occurring cells treated with 25 μM chloroquine for 1 hour. HeLa cells were co-stained with anti-LC3 (green) and anti-β-Tubulin (Red) antibodies and representative micrographs were shown. (**C**) Quantification of (A) and (B). Left and right panels show the LC3 puncta and the fold change per cell in each phase after chloroquine treatment respectively. *p < 0.05, **p < 0.01, ***p < 0.001. Scale bar, 10 μm.

To further analyze whether the autophagic flux was active during the different stages in naturally occurring mitosis, we did not use nocodazole or thymidine synchronization to avoid any potential perturbation. We used HeLa cells and compared the LC3 puncta in different stages with or without flux inhibitor. Here we used chloroquine because we wanted to validate the conclusions we got in Figure 6 by using NH_4_Cl. Our results show that although the LC3 puncta number was reduced in early phases of mitosis, which was consistent with previous findings [8, 9], chloroquine could accumulate autophagic structures in all stages of cell cycle, indicating that the autophagic flux was active in all stages during mitosis (Figure 7). It is interesting that chloroquine increased the LC3 puncta number to a greater extent in early stages of mitosis (prophase, prometaphase and metaphase) compared to interphase or the later stages of mitosis (telophase and cytokinesis) (Figure 7B–7C), which is consistent with our western blot results using NH_4_Cl as an autophagic flux inhibitor. These data demonstrate that the early stages of mitosis have higher autophagic flux compared to other mitotic phases.

### Differentially regulated autophagy regulators during cell cycle and upregulated Beclin-1 level in early mitosis

**Figure 8:**
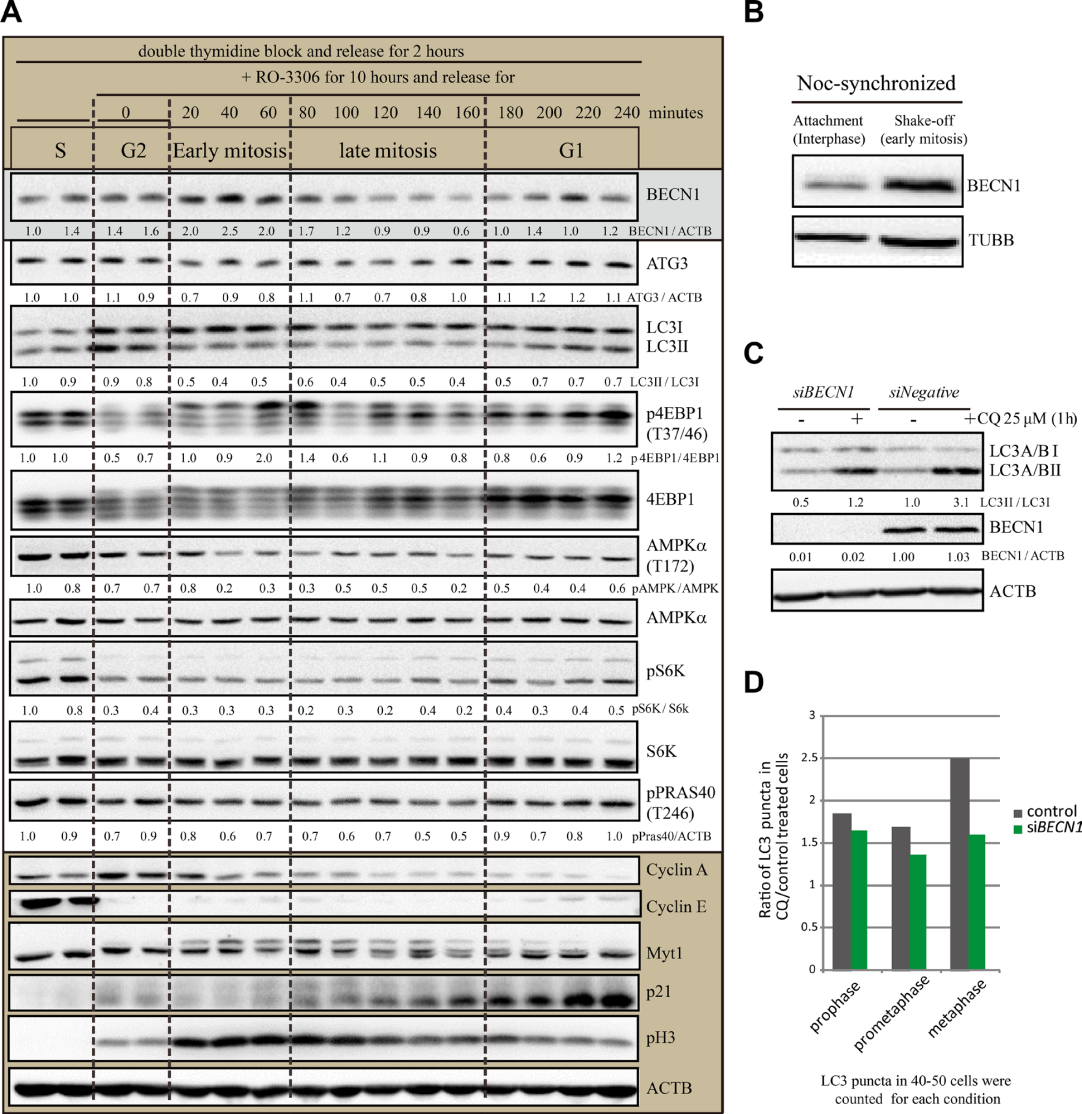
Differentially regulated autophagy regulators during cell cycle and upregulated Beclin-1 level in early mitosis (**A**) Western Blot analyzed the autophagy protein levels of HeLa cells released from double-thymidine with or without RO- 3306 synchronization. HeLa cells were released from double-thymidine for 2 hours before they were treated with RO-3306 for 10 hours. Then they were released for different timepoints. Densitometric analysis was performed and quantification results were labeled below the corresponding blots. Experiments were repeated for three times and representative results are shown. (**B**) Western blot results to compare Beclin-1 protein level in nocodazole-induced mitotic shake-off cells and the remaining attached interphase cells. HeLa cells were treated with double-thymidine block and released for 4 hours, added nocodazole for 7 hours before shake-off. (**C**) HeLa cells were transfected with Negative or *Beclin-1* siRNAs for 72 hours and treated with 25 μM chloroquine for 1 hour before cells were harvested and processed for Western blots. Representative results for anti-LC3 A/B, anti-Beclin-1 (BECN1) and anti-Actin (ACTB) were shown. (**D**) HeLa cells were transfected with Negative or *Beclin-1* siRNAs for 72 hours and treated with 25 μM chloroquine for 1 hour before cells were harvested and processed for immunofluorescence. 40–50 cells were counted for each condition.

To investigate the potential mechanism of the differential autophagic flux levels in the cell cycle, we analyzed some key autophagy regulators by western blots using double thymidine and RO-3306 synchronization (Figure 8). It is obvious that the MTOR substrate pS6K is downregulated in mitosis, which implies that MTOR is inhibited to induce autophagy. However, MTOR activity is also inhibited in late G2 phase, therefore the MTOR-induced autophagy is less likely to be the major reason that differentiates the autophagic flux difference between late G2 phase and early mitosis. In contrast, it is interesting that Beclin-1, MTOR and AMPK are all differentially regulated during the different cell cycle stages. Among them, Beclin-1 protein level is clearly higher in early mitosis than in other stages, including later mitosis. We also tested Beclin-1 protein level in nocodazole-induced pseudo-mitosis (prometaphase-like) (Figure 8B). Western Blot analysis in shake-off experiments showed that Beclin-1 protein level in mitosis (shake-off) was higher than interphase (attachment) cells. To test whether Beclin-1 functions in autophagy in mitosis, we used RNAi experiment to knock down *Beclin-1*, which reduced the LC3II/LC3I level in unsynchronized cells (Figure 8C), consistent with its known role in autophagy. Then we compared the chloroquine-induced LC3 puncta fold change in control and *Beclin-1* RNAi cells (Figure 8D). We counted LC3 puncta in 40–50 cells in each phase of early mitosis, including prophase, prometaphase and metaphase. It is clear that *Beclin-1* RNAi reduced chloroquine induced LC3 puncta increase in early mitosis (Figure 8D). This is consistent with the previously reported observation that Beclin-1 knockdown arrested cells in early mitosis [20]. Therefore the autophagic flux is differentially regulated during cell cycle stages and Beclin-1 may contribute to the highly active autophagic flux in early mitosis.

## DISCUSSION

It is very interesting that a similar question was raised about whether endocytosis persists during mitosis. It was suggested by some studies that endocytosis was shut down during mitosis, and only resumed in telophase and cytokinesis [23, 24]. However, some evidence showed that endocytosis was normal during mitosis [25]. This debate was resolved by Tacheva-Grigorova et al. in 2013 by doing detailed analysis to confirm that endocytosis is active throughout the cell division [26]. They found that people should pay attention to the chemical compounds used in the study because cells might behave abnormally after chemical perturbation, such as nocodazole. This is not very surprising because nocodazole depolymerizes microtubules, which are important for endocytosis, as well as other cellular processes such as autophagy.

A few studies suggested that autophagy was inhibited during mitosis. People thought that nuclear envelopes in mammalian cells are broken down so that their spindles, dividing chromosomes and fragmented Golgi are all exposed. Therefore it was likely that autophagy was controlled temporally and spatially during mitosis to avoid harming the exposed organelles. It had been proposed by Furuya et al. that autophagy was inhibited in early stages of mitosis because the inhibitory phosphorylation of VPS34 was increased in these stages [9]. However, their study also showed that VPS34 expression level was obviously increased in mitosis. Therefore whether phosphorylation of VPS34 is enough to completely inhibit its function is still not clear. In addition, it is also possible that different autophagy pathways are differentially regulated throughout the cell cycle so that inhibition of VPS34 alone is not sufficient to shut down autophagy in mitosis.

In fact, there were a few more studies that indicated that autophagy plays some roles in mitosis. In 2013, Matsui et al. showed that autophagy deficiency could cause abnormal mitosis [27]. In addition, Beclin-1 was shown to have a role in mitotic chromosome congression and outer kinetochore assembly [20] and ATG5 was shown to promote mitotic catastrophe [28]. Overexpression of ATG5 caused prolonged mitosis and unequally segregated chromosomes during mitosis. Additionally, Park et al. found that ATG5, Beclin-1 or UVRAG RNAi could all cause centrosome amplification [29], which usually lead to abnormal cell division and genome instability. Therefore these evidences also support our hypothesis that autophagy does play important roles in mitosis.

One novelty of our paper is that we used multiple cell lines and different methods to demonstrate that the autophagic flux in mitosis is indeed active. The decreased GFP-LC3 puncta in mitotic cells is not a proper marker to reflect the number of autophagic structure, nor autophagic flux level. As mentioned in the introduction, the first paper that directly addressed the question about autophagy in mitosis was in 2009 by Liu et al, who used ammonia as an autophagic flux inhibitor to show that the autophagic flux is active in mitosis [11]. Since ammonia also has autophagy inducing roles, here we used multiple autophagic flux inhibitors, bafilomycin A1 chloroquine and ammonia for shorter timepoints to test the dynamic mitotic process more accurately. In addition, Liu et al. analyzed synchronized cells by nocodazole, by which the autophagic flux may be potentially affected, but we examined both synchronized and naturally occurring mitotic cells. In addition, we also looked into more details about the different phases during mitosis, including prophase, prometaphase and metaphase, as well as other stages, such as G1, S and G2 phases. We used multiple assays to show that S phase and early mitotic cells have higher autophagic flux compared to late G2 phase, late mitosis as well as G1 phase. This is consistent with a previous report that resveratrol-induced S-G2/M cell cycle arrest is dependent on autophagy [30]. In addition, it has been proposed that cell growth and autophagy coordinate with each other [31] and the autophagy regulation mechanism is cell cycle dependent [17]. Although we found that Beclin-1 is upregulated in mitosis and its knockdown reduces autophagic flux in mitosis, further investigations of the detailed autophagy pathways at each cell cycle stage are strongly needed. In addition, it is likely that autophagy inhibitors would have more inhibition effects on actively dividing cells than normal cells. In fact, it has been shown that combination of autophagy inhibitors and chemodrugs that block cells in mitosis (such as Taxol and Vinblastine) could have better anti-tumor efficacy [32, 33].

In conclusion, our results indicated that the decreased autophagic structure number in early mitosis was not a result of autophagy inhibition, but a rapid clearance of autophagosomes. Our study resolved a literature debate by showing that mitotic cells have active autophagic flux. We showed that the autophagic flux is active throughout the cell cycle. More importantly, we show that early mitosis and S phase have relatively higher autophagic flux than G1 and late G2 phases, which might be helpful to degrade the damaged organelles and provide energy during S phase and mitosis.

## MATERIALS AND METHODS

### Antibodies and reagents

The autophagy antibody sampler kit, the cell cycle regulation antibody sampler kit II, the AMPK and ACC antibody sampler kit, the antibody for 4E-BP1(#9644), phospho-4E-BP1(T37/46)(#2855), SQSTM1(#5114), S6K (#2708), phospho-S6K(#9234), phospho-pras40(T246)(#2997), the HRP-linked anti-rabbit and anti-mouse IgG antibodies were all from Cell signaling technology. The anti-Cyclin B1 (GNS1, sc-245) antibody was acquired from Santa Cruz and anti-β-Tubulin, anti-GAPDH and anti-β-Actin antibodies from Beijing TransGen Biotech (Beijing, China). The GlutaMAX supplement and puromycin dihydrochloride were from Gibco. The secondary fluorescently conjugated antibodies, anti-fade prolong Gold with DAPI were from Molecular Probes. Pre-stained Protein Ladder (26616) and M-PER buffer were from Thermo Pierce. Bafilomycin A1 was from Cayman. Chloroquine, NH_4_Cl, RO-3306 and Thymidine were from Sigma. Nocodazole was from Selleckchem. Protease inhibitor and Phosphatase inhibitor cocktails were from Roche and the PVDF membrane from Millipore.

### Cell culture and generation of stable cell line

HeLa, 3T3, CHO and Hs578Bst cells were cultured in DMEM (without L-Glutamine) supplemented with 10% FBS, 2 mM GlutaMAX and 1% penicillin/streptomycin (P/S). HeLa-mCherry-EGFP-LC3 cells were established using retrovirus system. Retroviruses were packaged by transfecting the plasmid inserted the indicated genes with two helper plasmids into 293T cells using Fugene 6 (Promega) and the supernatant containing viruses was harvested after 48 hours. Stable cell line was generated by infection of HeLa using MSCV viruses and screened by 1 μg/ml puromycin and the stable cell lines were maintained in medium containing 1 μg/ml puromycin.

### Immunofluorescence

HeLa, NIH-3T3, CHO, Hs578Bst or HeLa-mCherry-EGFP-LC3 cells were grown on coverslips and treated with the drugs for indicated time points. Cells were washed once with PBS and fixed by −20°C methanol for 5 minutes and then blocked by AbDil-Tx (TBS-Tx supplemented with 2% BSA and 0.05% sodium azide) at room temperature for 30 minutes, followed by primary antibodies (anti-LC3A/B, anti-β-Tubulin and anti-GFP) incubation at 4°C overnight. The secondary fluorescently conjugated antibodies were incubated at room temperature for 1 hour and washed by TBS-Tx (TBS added with 0.1% Triton x-100) and mounted by anti-fade prolong Gold with DAPI. Images were taken using a Leica DMI4000B fluorescent microscope and the confocal microscope Zeiss LSM710. All experiments were repeated at least three times and representative micrographs are shown in the Figures.

### Live cell imaging of HeLa-mCherry-EGFP-LC3 cells

HeLa-mCherry-EGFP-LC3 cells were seeded into the Glass Bottom Culture Dishes (35 mm petri dish, 14 mm Microwell, MatTek Corporation) 24 hours before the experiment with DMEM (without phenol red and without L-Glutamine) supplemented with 10% FBS, 2 mM GlutaMAX and 1% penicillin/streptomycin (P/S). The live cells were then subjected to live cell imaging by the Leica microscope using the GFP and N2.1 channels.

### Western blot

Cells were lysed by M-PER buffer supplemented with protease and phosphatase inhibitor cocktails at 4°C for 20 minutes. The whole cell lysate was mixed with 5× SDS loading buffer thoroughly and denatured at 95°C for 5 minutes. The samples were subjected to the SDS-PAGE in Bio-Rad Mini-PROTEAN Tetra Cell and transferred by Thermo Scientific Owl VEP-2 (7351). The PVDF membrane was blocked with 5% NFDM (non-fat dried milk) at room temperature for 30 minutes and the corresponding primary and HRP-conjugated secondary antibodies. Western Blot results were obtained by Tanon *Fine-do* X6 (Shanghai, China). ImageJ software was used to quantify the protein relative levels shown by Western Blot. Mean values are shown in the Figures, and SDs are shown as error bars. All experiments were repeated for at least three times and representative results were shown in the Figures.

### RNAi assay

HeLa cells were plated in 12-well or 24-well plate and 40 nM siRNAs for *Beclin-1* or negative control were transiently transfected using Hiperfect following manufacture's protocol. The siRNA sequence for *Beclin-1* was CAGUUUGGCACAAUCAAUA. After 72 hours incubation, the cells were either lysed for Western Blot or fixed for immunofluorescence experiments.

### Cell synchronization

HeLa cells were plated in 35-mm-dish at 25% confluence 24 hours before double-thymidine block assay. Cells were firstly blocked with 2.5 mM Thymidine in DMEM for 16 hours and then released for 8 hours in fresh DMEM medium before washing with warm PBS twice. Then a second thymidine block for another 16 hours was performed to capture cells to G1/S border. Alternatively, nocodazole (100 ng/ml) was combined with double-thymidine block to get pseudo-prometaphase cells. Shake-off was used to separate mitotic and interphase cells. Moreover, cells released from double-thymidine block for 2 hours were treated with RO-3306 (10 μM) for another 10 hours to acquire late G2-phase cells. After washing twice with warm PBS, cells progress through mitosis sequentially. Cells in different stages were lysed to performed SDS-PAGE and Western Blot or fixed for immunofluorescence.

### Cell cycle analysis by FACS

HeLa cells blocked by double-thymidine were released in fresh DMEM complete medium at different time points and trypsinized with 0.25% Trypsin/EDTA. After washing with ice-cold PBS twice, cells were fixed with −20°C 70% ethanol overnight and then marked with PI (propidium iodide) solution (BD pharmingen) as the manufacturer's protocol indicated. The raw data about cell cycle distribution were acquired by BD Calibur flow cytometry and analyzed by Modfit LT software.

### Semi-quantitative RT-PCR and Real-time RT- PCR analysis

RNAs were isolated from HeLa cells released from double-thymidine block at different time points. Semi-quantitative RT-PCR and real-time RT-PCR were performed as previously described [34, 35]. The primers used for semi-quantitative RT-PCR were as follows:

ATG3F:GGATGGGTAGATACATATCACAACACAG,

ATG3R: CATATTCTATTGTTGGAATGACAGCTTG;

ATG5F: AAAGTGAAAAAGCACTTTCAGAAGGTTA,

ATG5R: AATATGAAGAAAATTATCCGGGTAGCTC;

BECLIN1F: GATACTCTTTTAGACCAGCTGGACACTC,

BECLIN1R: ATTTGTCTGTCAGAGACTCCAGATATGA;

LC3BF: CATCAAGATAATTAGAAGGCGCTTACAG,

LC3BR: CCATCTTCATCTTTCTCACTCTCATACA;

SQSTM1F: GGATGATGAGGAGAATTACTTGGATTTA,

SQSTM1R: CGTAACTTTGTTCTGCGTATGTATCATC;

CCNBF: GACTGGCTAGTACAGGTTCAAATGAAAT,

CCNBR: GTTCTTGACAGTCATGTGCTTTGTAAGT.

The primers used for real-time RT-PCR were as follows:

GAPDH-Forward: TCCACTGGCGTCTTCACC,

GAPDH-Reverse: GGCAGAGATGATGACCCTTTT;

LC3B (MAP1LC3B) -Forward: CGCACCTTCGAACA AAGAG,

LC3B (MAP1LC3B) -Reverse: CTCACCCTTGTATCG TTCTATTATCA;

Beclin-1(BECN1)-Forward: GGATGGTGTCTCTCGCAGAT,

Beclin-1(BECN1)-Reverse: TTGGCACTTTCTGTGGACAT;

CyclinB1 (CCNB1)-Forward: AAGAGCTTTAAACTTT GGTCTGGG,

CyclinB1 (CCNB1)-Reverse: CTTTGTAAGTCCTTGA TTTACCATG.

Real-time PCR assay was performed in Lightcycler 96 (Roche) and 10 μl reaction system was used. Briefly, 5 μl SYBR Green I Master(2×), 0.3 μl forward primer and 0.3 μl reverse primer, 2 μl cDNA and 2.4 μl distilled water were mixed gently (10 μl reaction system) and performed in Lightcycler 96 (Roche). The programs were run as follows: 95°C, 600s; 45 cycles for (95°C, 10s; 60°C, 15s; 72°C, 15s); 95°C, 10s; 65°C, 60s; 97°C, 1s.

### Statistical analysis

ImageJ software was used to quantify the protein relative levels shown by Western Blot and Graphpad prism 5 was used to analyze the data using One-way and Two-way ANOVA. The micrographs acquired by Zeiss LSM710 were split and merged by ImageJ software and cropped by Adobe Photoshop.

## SUPPLEMENTARY MATERIALS FIGURES


