# Supplementary material for: Autophagic flux is highly active in early mitosis and differentially regulated throughout the cell cycle
Source: Oncotarget. 2016 May 18;7(26):39705–18. doi: 10.18632/oncotarget.9451 (PMC5129964; doi:10.18632/oncotarget.9451)
Supplement: Supplementary file 1 [file oncotarget-07-39705-s001.pdf]

Autophagic flux is highly active in early mitosis and differentially regulated throughout the cell cycle

Supplementary Materials

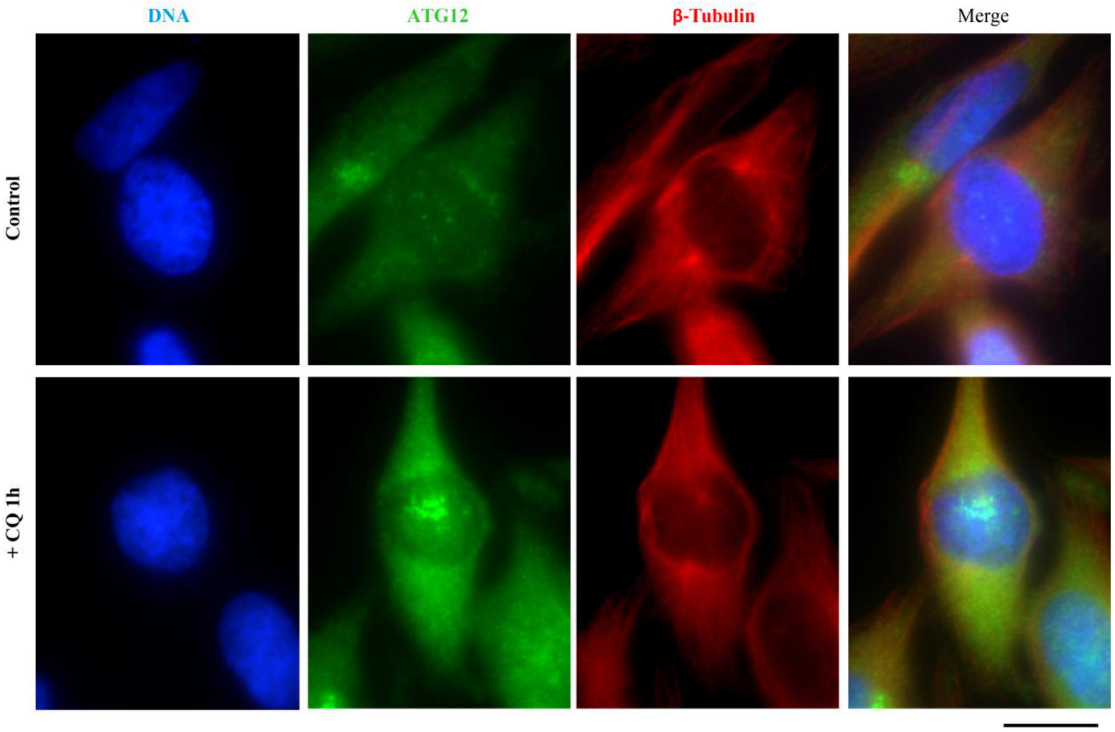

Supplementary Figure S1: ATG12 staining in HeLa cells with or without chloroquine treatment. HeLa cells were treated with control or 25 μM chloroquine (CQ) for 1 hour before they were fixed and stained with anti-β-Tubulin (red) and anti-ATG12 (green) antibodies and DAPI (blue). Scale bar: 10 μm.

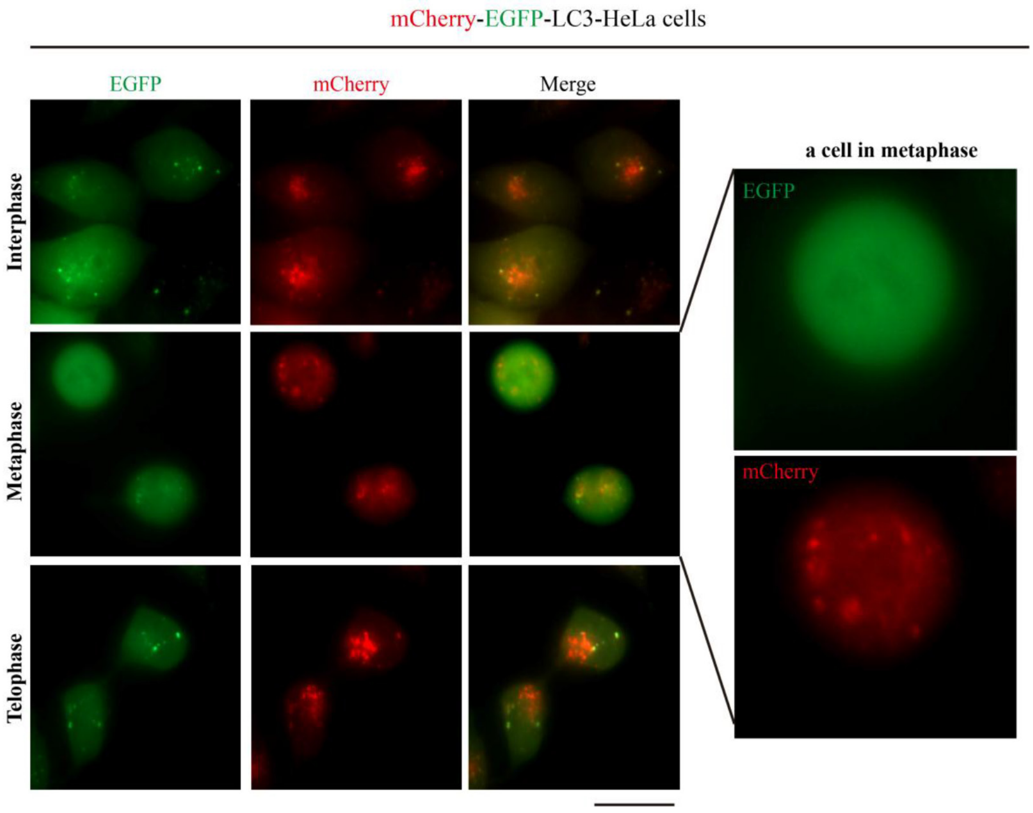

Supplementary Figure S2: mCherry-EGFP-LC3-HeLa cells in different cell cycle stages. mCherry-EGFP-LC3-HeLa cells were imaged without fixation. Scale bar: 10 μm. Right panel shows enlarged images of one of the metaphase cells from the left.

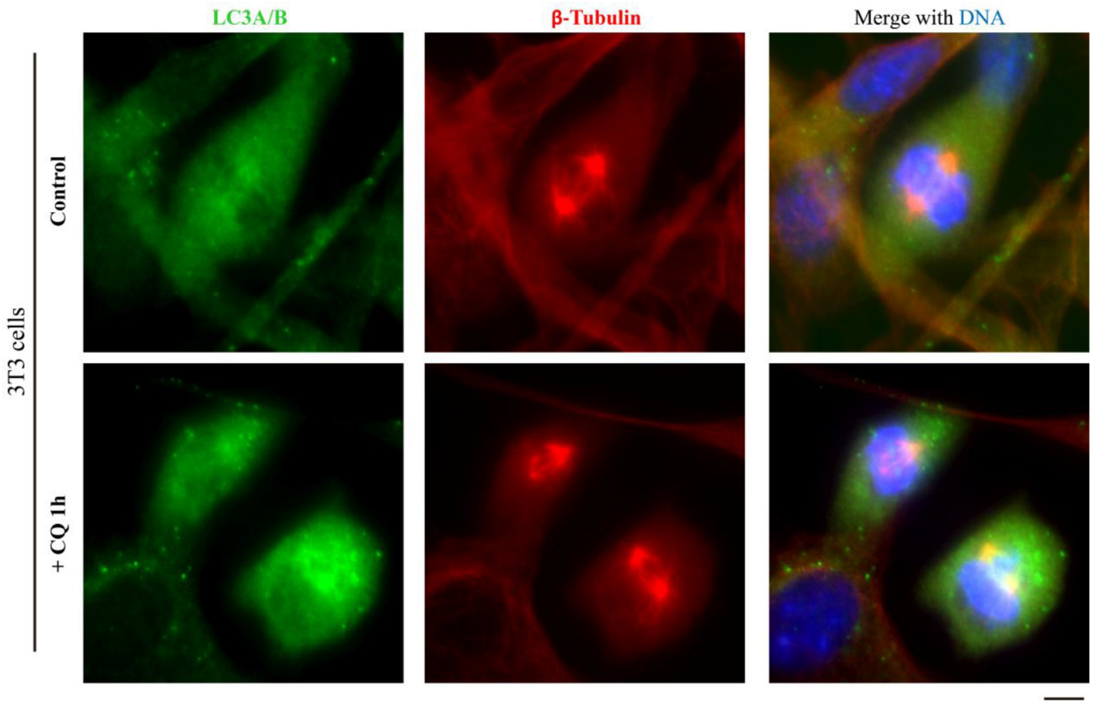

Supplementary Figure S3: Autophagy is active in mitotic 3T3 cells. 3T3 cells were treated with control or 25 μM chloroquine (CQ) for 1 hour before they were fixed and subjected to immunofluorescence experiment. Anti-β-Tubulin (red), anti-LC3 A/B (green) antibodies and DAPI (blue) were used. Scale bar, 10 μm.

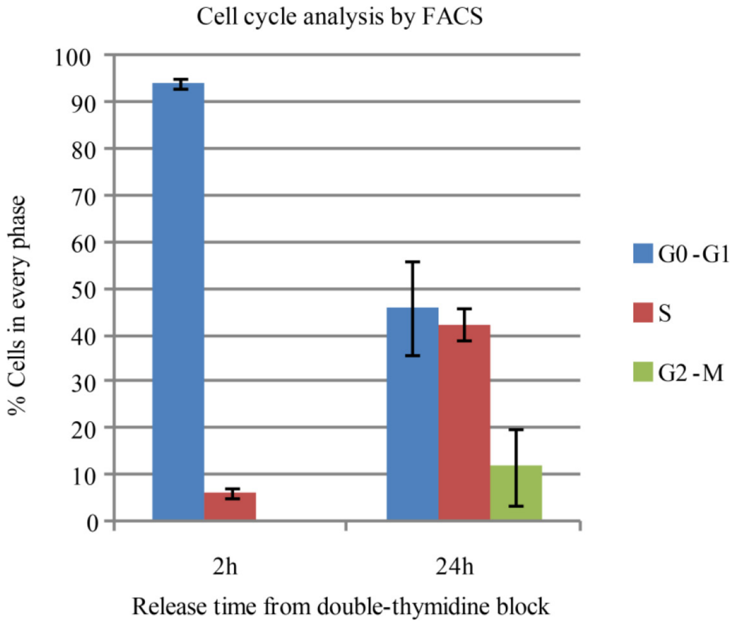

Supplementary Figure S4: Quantification of FACS results of HeLa cells 2 hours and 24 hours after double-thymidine block. *n* = 3.
